# Bidirectional relationship between subjective age and frailty: a prospective cohort study

**DOI:** 10.1186/s12877-021-02344-1

**Published:** 2021-06-29

**Authors:** Yuxiao Li, Minhui Liu, Christina E. Miyawaki, Xiaocao Sun, Tianxue Hou, Siyuan Tang, Sarah L. Szanton

**Affiliations:** 1 Xiangya School of Nursing, Central South University, 172 Tongzipo Road of Yuelu District, Hunan 410013 Changsha, China; 2 School of Nursing, Johns Hopkins University, Baltimore, MD USA; 3 Graduate College of Social Work, University of Houston, Houston, TX USA

**Keywords:** Subjective age, Frailty, Older adults

## Abstract

**Background:**

Subjective age refers to how young or old individuals experience themselves to be and is associated with health status, behavioral, cognitive, and biological processes that influence frailty. However, little research has examined the relationship between subjective age and frailty among older adults. This study examined the bidirectional association between subjective age and frailty among community-dwelling older adults.

**Methods:**

We used data from the 2011 to 2015 waves of the National Health and Aging Trends Study. Our sample consists of 2,592 community-dwelling older adults with complete data on main outcome variables. Subjective age was measured by asking participants, “What age do you feel most of the time?” Based on the five phenotypic criteria: exhaustion, unintentional weight loss, low physical activity, slow gait, and weak grip strength, frailty was categorized into robust = 0, pre-frailty = 1 or 2; frailty = 3 or more criteria met. Generalized estimating equation models were used to examine the concurrent and lagged association between subjective age and frailty.

**Results:**

Participants were, on average, 75.2 ± 6.8 years old, non-Hispanic whites (76 %), female (58 %). 77 % of the participants felt younger, 18 % felt the same, and 5 % felt older than their chronological age. About 45 %, 46 %, and 9 % of the participants were robust, pre-frailty and frailty in the first wave, respectively. Generalized estimating equations revealed that an “older” subjective age predicted a higher likelihood of pre-frailty and frailty (OR, 95 % CI = 1.93, 1.45–2.56).

**Conclusions:**

These findings suggest that people with older subjective age are more likely to be pre-frail/frail. Subjective age could be used as a quick and economical screening for those who are potentially frailty or at risk for frailty.

## Background

Older adults with frailty are at a greater risk of adverse outcomes such as falls, disability, hospitalization or institutionalization and mortality [1–4]. Frailty is a dynamic and reversible process that can be delayed by targeted interventions such as exercise and nutrition [5–7]. Therefore, it is important to identify factors associated with frailty.

Age is an important risk factor for frailty because increased chronological age predicts frailty [8]. Subjective age – how old a person perceives themselves to be, depending on an individual’s self-assessment of the degree of aging - is another concept that may influence people’s aging. Feeling younger than one’s chronological age is considered a protective factor to buffer against the negative effects of aging, such as old-age stereotypes and social stigma [9–11]. Subjective age has been considered as a biopsychosocial marker of aging that can predict an individual’s health condition [12, 13]. A large body of literature has shown that older subjective age is associated with a series of negative health outcomes, including poorer mental health [14], worse physical, functional and cognitive health [15–18], increased risk of hospitalizations [19] and reduced longevity [20, 21]. Despite the significance of subjective age in people’s health, to our knowledge, little research has examined the relationship between subjective age and frailty among older adults.

There are reasons to expect that subjective age may be related to frailty, given that subjective age is associated with health status, behavioral, cognitive, and biological processes that influence frailty. People with a younger subjective age have a better physical function, including higher scores for activities of daily living (ADL) or instrumental activities of daily living (IADL) [22, 23], stronger grip strength [24] and faster walking speed [16]. Subjective age is also a significant predictor of many psychological factors among older adults. Previous studies have shown that individuals with younger subjective age tend to have better mental health [25, 26]. Specifically, younger subjective age was associated with fewer depressive symptoms [26], less stress [12] and less loneliness [27]. Previous studies have found that younger subjective age is associated with better cognitive functions [28]. Older adults who felt younger than their chronological age showed better long-term memory performance and executive function 10 years later, even after adjusting for chronological age and other demographic and health variables [18]. Frailty is multifactorial in etiology, and physical, psychological and cognitive functions are all risk factors of frailty among older adults [29]. Therefore, subjective age may be a marker of frailty. Frail older adults may feel older than their chronological age, because weakness, exhaustion, slow gait, and low physical activity are part of frailty syndrome [17, 30].

The purpose of this study was to examine the bidirectional relationships between subjective age and frailty. We hypothesized that (1) older adults with an older subjective age would be at higher risk of being pre-frail/frail; and (2) older adults with pre-frailty/frailty tend to feel older than their chronological age.

## Methods

### Study sample

We used data from 2011 to 2015 waves of the National Health Aging Trends Study (NHATS), a nationally representative longitudinal cohort study that has collected a sample of Medicare beneficiaries ages 65 or older in the United States. Our study sample consists of 2,592 community-dwelling older adults with complete data on main outcome variables (subjective age and frailty) from 2011 to 2015 waves. Compared to participants who were included in this study, the excluded participants felt older, had less education, and had more ADL/IADL impairment, chronic diseases, hospitalizations, and falls with poorer health status. The excluded participants were also less obese, engaged in less vigorous activity, but more demented. The NHATS was approved by the Johns Hopkins Bloomberg School of Public Health IRB. NHATS participants completed written informed consent prior to being interviewed.

### Measurements

#### Dependent variable

Frailty was assessed using the modified frailty phenotype paradigm [31] based on five criteria: unintentional weight loss, exhaustion, weakness, slow gait and low physical activity. Criteria were operationalized from NHATS interviews and performance assessments. (1) unintentional weight loss: involuntarily losing 10 pounds or more in the last year; (2) exhaustion: self-reported low energy or being easily exhausted to limiting activities; (3) weakness: grip strength measured by dominant hand over 2 trials as being at or below the 20th percentile within eight sex-by-body mass index (BMI) categories; (4) slow gait: gait speed from the best of two timed 3-meter walk tests being at or below the 20th percentile within four sex-height categories; and (5) low physical activity: self-reported not having taken part in vigorous activities or never walked for exercise in the last month. Participants who met three or more criteria were considered as “frailty.” Those with one or two criteria were considered as “pre-frailty,” and those without any criterion as “robust.”

#### Independent variables

Subjective age was measured by the question, “Sometimes people feel older or younger than their age. During the last month, what age did you feel most of the time?” Participants answered a number in years to estimate the age they felt. We calculated proportional discrepancy scores by subtracting chronological age from felt age and divided by chronological age [32]. A negative score indicates a younger subjective age, while a positive score indicates an older subjective age. We coded equal = 0, younger subjective age = 1, older subjective age = 2. Values three standard deviations above or below the mean were considered outliers and excluded from the analysis.

#### Covariates

Demographic characteristics included chronological age; sex (coded 0 for female and coded 1 for male); race/ethnicity (coded 0 to 4 for “non-Hispanic White, non-Hispanic Black, Indian/Asian/Native/Hawaii, Hispanic, other”); education (coded 0 to 3 for “less than high school, high school graduates, some college or vocational school, bachelor or higher”); living arrangement (coded 0 to 3 for “alone, with spouse/partner only, with others only, with spouse/partner and with others”).

Health-related variables included the following: (1) bothersome pain was measured by the question “In the last month, have you been bothered by pain?” (0 = no; 1 = yes); (2) depressive symptoms, assessed by the Patient Health Questionnaire-2 (PHQ-2), which measured how often the participant had been bothered by (a) “little interest or pleasure in doing things” and (b) “feeling down, depressed or hopeless” over the last month. Responses were on a 4-point Likert scale (0 = not at all; 1 = several days; 2 = more than half the days; 3 = nearly every day). We summed scores on both of the PHQ-2 questions to create a score from 0 to 6, with scores > 3 were classified as depressive symptom (0 = no; 1 = yes); (3) ADL impairments, we computed the number of activities (eating, dressing, bathing and toileting) in which participants had any difficulty in the past month; (4) IADL impairments, we calculated the number of activities (doing laundry, shopping, preparing meal, managing money and taking medication) in which participants had difficulty in the past month; (5) body mass index (BMI) (coded 0 for normal with BMI < 30 kg/m^2^ and coded 1 for obesity with a BMI ≥ 30 kg/m^2^); (6) self-rated health status (code 0 to 4 for “excellent, very good, good, fair, poor”); (7) the number of chronic illnesses (high blood pressure, heart attack/heart disease, arthritis, osteoporosis, diabetes, lung disease, stroke, and cancer) diagnosed by a doctor (coded 0 for no disease, coded 1 for have 1–3 diseases and coded 2 for have more than 4 diseases); (8) hospitalized in the last 12 months (0 = no; 1 = yes); (9) fall was measured with the question “In the past 12 months, have you fallen down?” (0 = no; 1 = yes); (10) smoking (coded 0 for never smokers and coded 1 for current/former smokers); (11) dementia was assessed by asking participants to report whether they had ever been diagnosed by a doctor with dementia or Alzheimer’s disease (0 = no; 1 = yes); (12) vigorous activities, was assessed by asking participants to report whether they ever spend time on vigorous activities that increased their heart rate and made them breathe harder in the last month (0 = no; 1 = yes).

### Statistical analyses

Descriptive statistics were presented as mean ± SD (standard deviation) for continuous variables or absolute number and percentage for categorical variables. Chi-square tests or ANOVAs were used to compare the baseline characteristics of the sample according to subjective age categories (younger than chronological age, equal to chronological age, and older than chronological age) and frailty categories (robust, pre-frailty, and frailty). We used generalized estimating equation (GEE) models to test the concurrent and lagged association between subjective age and frailty. GEE is an extension of the generalized linear model that accounts for the within-subject correlation across repeated measurements and is appropriate to estimate population-averaged effects over time [33]. Since missing values on covariate variables were less than 1 %, we did not apply any techniques to handle them.

#### Subjective age as a predictor of pre-frailty or frailty

We estimated two sets of GEE models, specifying the logit link function with a binomial distribution [34, 35]. We assumed an exchangeable correlation structure and used robust standard errors to account for the correlation between measures for each measure. First, we assessed the concurrent association between subjective age and frailty. Subjective age at wave *w* was related to frailty at wave *w* with adjustment for frailty at wave *w-1*. The adjustment for frailty at wave *w-1* was made to account for the recurrent frailty and compensate for our inability to adjust for frailty history. Then, lagged GEE models were analyzed, in which subjective age at wave *w* was related to frailty at wave *w + 1* with adjustment for frailty at wave *w*. We presented the crude associations initially (Model 1), then we adjusted for demographics (chronological age, sex, race, education, and living arrangement) (Model 2), and finally further adjusted for health-related variables (pain, depression, number of ADL/IADL impairments, BMI, health status, number of chronic illnesses, hospitalized, fall, and smoking) (Model 3).

#### Pre-frailty or frailty as predictors of subjective age

We estimated another two sets of GEE models, specifying the identity link function with a gaussian distribution. The modeling strategy was similar to the analysis of the first two sets of GEE models. First, we assessed the concurrent association between frailty at wave *w* and subjective age at wave *w* with adjustment for subjective age at wave *w-1*. Then, lagged GEE models were analyzed, in which frailty at wave *w* was related to subjective age at wave *w + 1* with adjustment for subjective age at wave *w*.

All *P* values were two-sided and statistical significance was determined at *p* < 0.05. Statistical analyses were conducted using STATA 15.

## Results

A total of 2,592 community-dwelling older adults were included. 58 % were female and 76 % were non-Hispanic whites. The average chronological ages were 75.2 ± 6.8 years old. At baseline, a majority (77 %) of the participants felt younger than their chronological age, 18 % felt the same, and 5 % felt older. Baseline frailty assessments grouped participants as robust (45 %), pre-frailty (46 %), and frailty (9 %). The robust group (-0.20 ± 0.16), pre-frailty group (-0.16 ± 0.17), and frailty group (-0.10 ± 0.20) all showed a young subjective age (not shown in tables).

### Subjective age as a predictor of pre-frailty or frailty

Participants with older subjective age were more likely to be younger, less educated, reported more pain and depression, were obese, had higher numbers of ADL/IADL impairments and chronic illnesses, more often hospitalized, had more falls in the last year and less vigorous activities compare to those whose subjective ages were equal and younger (Table 1).

**Table 1:** Baseline characteristics of participants by subjective age categories (*N* = 2,592)

Characteristics	Total	Equal (*n* = 462)	Younger (*n* = 1,989)	Older (*n* = 141)	*p* values
Age, years, mean (SD)	75.2 (6.8)	75.4 (6.7)	75.3 (6.8)	73.4 (6.0)	**0.004**
Sex (Female), n (%)	1,495 (57.7)	269 (58.2)	1,139 (57.3)	87 (61.7)	0.568
Race, n (%)					0.693
White, non-Hispanic	1,976 (76.2)	337 (72.9)	1,536 (77.2)	103 (73.0)	
Black, non-Hispanic	458 (17.7)	95 (20.6)	334 (16.8)	29 (20.6)	
Indian/Asian/Native/Hawaii	48 (1.9)	8 (1.7)	37(1.9)	3 (2.1)	
Hispanic	105 (4.0)	21 (4.6)	78 (3.9)	6 (4.3)	
Other	5 (0.2)	1 (0.2)	4 (0.2)	0	
Education, n (%)					**< 0.001**
Less than high school	484 (18.7)	98 (21.2)	345 (17.4)	41 (29.1)	
High school graduates	691 (26.7)	148 (32.0)	498 (25.0)	45 (31.9)	
College or vocational school	673 (25.9)	103 (22.3)	535 (26.9)	35 (24.8)	
Bachelor or higher	744 (28.7)	113 (24.5)	611 (30.7)	20 (14.2)	
Living arrangement, n (%)					**0.013**
Alone	821 (31.8)	165 (35.7)	621 (31.3)	35 (25.0)	
With spouse/partner only	1,218 (47.1)	193 (41.8)	961 (48.5)	64 (45.7)	
With others only	308 (11.9)	56 (12.1)	225 (11.4)	27 (19.3)	
With spouse/partner and others	237 (9.2)	48 (10.4)	175 (8.8)	14 (10.0)	
Pain, n (%)	1,408 (54.3)	291 (63.0)	1,013 (50.9)	104 (73.8)	**< 0.001**
Depressive symptoms, n (%)	262 (10.1)	60 (13.1)	156 (7.8)	46 (32.9)	**< 0.001**
Number of ADLs, mean (SD)	0.3 (0.7)	0.4 (0.8)	0.2 (0.6)	0.8 (1.2)	**< 0.001**
Number of IADLs, mean (SD)	0.6 (1.1)	0.8 (1.3)	0.4 (1.0)	1.4 (1.7)	**< 0.001**
BMI ≥ 30 kg/m^2^, n (%)	807 (31.3)	180 (39.4)	560 (28.3)	67 (47.9)	**< 0.001**
Health status, n (%)					**< 0.001**
Excellent	413 (15.9)	32 (6.9)	379 (19.0)	2 (1.4)	
Very good	852 (32.9)	110 (23.9)	719 (36.1)	23 (16.3)	
Good	826 (31.9)	189 (41.0)	596 (30.0)	41 (29.1)	
Fair	392 (15.1)	99 (21.5)	244 (12.3)	49 (34.8)	
Poor	108 (4.2)	31 (6.7)	51 (2.6)	26 (18.4)	
Number of chronic illnesses, mean (SD)	2.4 (1.5)	2.8 (1.5)	2.3 (1.5)	3.3 (1.6)	**< 0.001**
Hospitalized, n (%)	484 (18.7)	96 (20.8)	347 (17.5)	41 (29.1)	**0.001**
Fall, n (%)	707 (27.3)	144 (31.2)	501 (25.2)	62 (44.0)	**< 0.001**
Smoking, n (%)	1,324 (51.1)	233 (50.4)	1,009 (50.7)	82 (58.2)	0.223
Dementia, n (%)	33 (1.3)	8 (1.7)	22 (1.1)	3 (2.1)	0.362
Vigorous activities, n (%)	1,151 (44.4)	168 (36.4)	950 (47.8)	33 (23.4)	**< 0.001**

*SD*Standard Deviation, *ADL *Activities of Daily Living, *IADL *Instrumental Activities of Daily Living, *BMI *Body Mass Index

Table 2 shows the results of GEE models using subjective age to predict frailty. The concurrent association between subjective age and frailty was significant in the unadjusted models (OR, 95 % CI = 3.87, 3.02–4.97). In the fully adjusted model (Model 3), adjustments for demographic and health-related covariates reduced the strength of these associations but did not fully attenuate them (OR, 95 % CI = 2.39, 1.76–3.24). Same as the concurrent associations, the lagged associations between subjective age and frailty were also significant in the unadjusted models (OR, 95 % CI = 2.50, 1.96–3.20). Older subjective age remained a significant independent predictor of subsequent frailty after controlling for demographic and health-related covariates (OR, 95 % CI = 1.88, 1.42–2.50).

**Table 2:** Generalized estimating equation analysis of subjective age predicting frailty

Models	Concurrent association^a^	Lagged association^a^
Model 1		
Subjective age	3.87 (3.02–4.97) ^***^	2.50 (1.96–3.20)^***^
Model 2		
Subjective age	4.23 (3.25–5.52) ^***^	2.59 (2.00-3.35) ^***^
Model 3		
Subjective age	2.39 (1.76–3.24) ^***^	1.88 (1.42–2.50) ^***^

Model 1: independent variable of interest

Model 2: Model 1 + demographic covariates (chronological age, sex, race, education, living arrangement)

Model 3: Model 2 + health-related covariates (pain, depression, number of ADLs/IADLs, BMI, health status, number of chronic illnesses, hospitalized, fall, smoking, dementia, vigorous activities)

^a^Odds ratio and 95 % confidence interval were reported here

^***^p < 0.001

### Pre-frailty or frailty as predictors of subjective age

Participants who were considered as frail tend to be older, less educated, non-Hispanic white, and female who lives alone. They reported significantly more pain, dementia and depression, higher numbers of ADL/IADL impairments and chronic illnesses, were obese, reported worse health status, significantly high percentages of hospitalization, had falls in the last year and less vigorous activities compared to those who were in the robust and pre-frail categories (Table 3).

**Table 3:** Baseline characteristics of participants by frailty categories (*N* = 2,592)

Characteristics	Total	Robust (*n* = 957)	Pre-frail (*n* = 1,228)	Frail (*n* = 407)	*P* values
Age, years, mean (SD)	75.2 (6.8)	74.1 (6.5)	76.0 (6.8)	76.6 (7.1)	**< 0.001**
Sex (Female), n (%)	1,495 (57.7)	611 (52.5)	732 (61.1)	152 (66.4)	**< 0.001**
Race, n (%)					**< 0.001**
White, non-Hispanic	1,976 (76.2)	942 (80.9)	907 (75.7)	127 (55.5)	
Black, non-Hispanic	458 (17.7)	161 (13.8)	223 (18.6)	74 (32.3)	
Indian/Asian/Native/Hawaii	48 (1.9)	23 (2.0)	21 (1.8)	4 (1.8)	
Hispanic	105 (4.1)	39 (3.3)	43 (3.6)	23 (10.0)	
Other	5 (0.2)	0	4 (0.3)	1 (0.4)	
Education, n (%)					**< 0.001**
Less than high school	484 (18.7)	137 (11.8)	260 (21.7)	87 (38.0)	
High school graduates	691 (26.7)	275 (23.6)	359 (30.0)	57 (24.9)	
College or vocational school	673 (26.0)	296 (25.4)	320 (26.7)	57 (24.9)	
Bachelor or higher	744 (28.7)	457 (39.2)	259 (21.6)	28 (12.2)	
Living arrangement, n (%)					**< 0.001**
Alone	821 (31.8)	346 (29.8)	395 (33.1)	80 (35.2)	
With spouse/partner only	1,218 (47.1)	637 (54.8)	507 (42.4)	74 (32.6)	
With others only	308 (11.9)	100 (8.6)	161 (13.5)	47 (20.7)	
With spouse/partner and others	237 (9.2)	79 (6.8)	132 (11.0)	26 (11.5)	
Pain, n (%)	1,408 (54.3)	470 (40.3)	749 (62.5)	189 (82.5)	**< 0.001**
Depressive symptoms, n (%)	262 (10.1)	51 (4.4)	147 (12.3)	64 (28.2)	**< 0.001**
Number of ADLs, mean (SD)	0.3 (0.7)	0.08 (0.3)	0.3 (0.7)	1.2 (1.3)	**< 0.001**
Number of IADLs, mean (SD)	0.6 (1.1)	0.2 (0.5)	0.7 (1.1)	2.0 (1.8)	**< 0.001**
BMI ≥ 30 kg/m^2^, n (%)	807 (31.4)	290 (25.0)	422 (35.5)	95 (42.0)	**< 0.001**
Health status, n (%)					**< 0.001**
Excellent	413 (15.9)	288 (24.7)	120 (10.0)	5 (2.2)	
Very good	852 (32.9)	488 (41.9)	340 (28.4)	24 (10.5)	
Good	826 (31.9)	321 (27.6)	438 (36.6)	67 (29.2)	
Fair	392 (15.1)	62 (5.3)	243 (20.3)	87 (38.0)	
Poor	108 (4.2)	6 (0.5)	56 (4.7)	46 (20.1)	
Number of chronic illnesses, mean (SD)	2.4 (1.5)	1.9 (1.3)	2.7 (1.5)	3.5 (1.6)	**< 0.001**
Hospitalized, n (%)	484 (18.7)	127 (11.0)	281 (23.5)	76 (33.3)	**< 0.001**
Fall, n (%)	707 (27.3)	222 (19.1)	373 (31.2)	112 (49.1)	**< 0.001**
Smoking, n (%)	1,324 (51.1)	588 (50.5)	612 (51.1)	124 (54.2)	0.596
Dementia, n (%)	33 (1.3)	9 (0.8)	17 (1.4)	7 (3.1)	**0.016**
Vigorous activities, n (%)	1,151 (44.4)	766 (65.8)	366 (30.6)	19 (8.3)	**< 0.001**

*SD *Standard Deviation, *ADL *Activities of Daily Living, *IADL *Instrumental Activities of Daily Living, *BMI *Body Mass Index

Table 4 presents the results of GEE analysis using frailty status to predict subjective age. The concurrent association between frailty and subjective age was significant, even after adjusting demographic and health-related variables (Exponentiated coefficient, 95 % CI = 1.014, 1.007–1.020). The 5-year lagged associations between subjective age and frailty were significant in the unadjusted models (Exponentiated coefficient, 95 % CI = 1.007, 1.002–1.012) and partially adjusted models (Exponentiated coefficient, 95 % CI = 1.008, 1.003–1.013). However, after adjusting for health-related variables, the association between frailty and subjective age became insignificant. (Exponentiated coefficient, 95 % CI = 1.004, 0.998–1.010).

**Table 4:** Generalized estimating equation analysis of frailty predicting subjective age

Models	Concurrent association^a^	Lagged association^a^
Model 1		
Frailty		
Robust (reference group)	1.00	1.00
Pre-frailty and frailty	1.019 (1.014–1.024) ^***^	1.007 (1.002–1.012) ^**^
Model 2		
Frailty		
Robust (reference group)	1.00	1.00
Pre-frailty and frailty	1.021 (1.016–1.026) ^***^	1.008 (1.003–1.013) ^**^
Model 3		
Frailty		
Robust (reference group)	1.00	1.00
Pre-frailty and frailty	1.014 (1.007–1.020) ^***^	1.004 (0.998–1.010)

Model 1: independent variable of interest

Model 2: Model 1 + demographic covariates (chronological age, sex, race, education, living arrangement)

Model 3: Model 2 + health-related covariates (pain, depression, number of ADLs/IADLs, BMI, health status, number of chronic illnesses, hospitalized, fall, smoking, dementia, vigorous activities)

^a^Exponentiated coefficient and 95 % confidence interval were reported here

**p < 0.01; ***p < 0.001

## Discussion

Using a nationally representative sample of community-dwelling older adults, we found that subjective age independently predicted frailty, even after accounting for demographics and health-related factors. People with older subjective age were more likely to be pre-frailty/frailty.

To our knowledge, there has been one study that examined the relationship between subjective age and frailty, using cross-sectional data from a longitudinal cohort [36]. Contrary to our study, they found no significant association between subjective age and frailty. Possible reasons for these inconsistent findings could be the different samples (nursing home residents vs. community-dwelling older adults), sample sizes (*N* = 272 vs. 2,592), and data analyzed (cross-sectional vs. longitudinal). For example, their study had a relatively small sample size but used a three-categorical frailty status outcome variable. It might not have sufficient statistical power to detect the significant associations between subjective age and frailty. However, their study also included measures that assessed older adults’ attitudes toward their aging process and found that frailty nursing home residents had a more negative attitude toward one’s own aging compared to pre-frail and robust residents. This partially supports our study findings.

Our findings suggested that feeling older than one’s chronological age was associated with a higher risk of frailty. To our knowledge, there is no previous longitudinal evidence that subjective age is associated with frailty. However, previous studies have shown that having an older subjective age is a risk factor for outcomes of lower walking speed, weaker grip strength, and increased difficulty with ADLs [17, 22, 24]. Given that some of these outcomes are part of frailty diagnostic criteria, a bidirectional association between subjective age and frailty could be expected and was confirmed in our study. In addition, our results showed that the association between subjective age and frailty is significantly reduced after adjusting for health-related variables. This result suggests that health-related variables may partly mediate the association between older subjective age and a higher likelihood of frailty. Further research is needed to identify which health-related variables are at work and the mechanisms of mediation paths between subjective age and frailty.

Unlike our hypothesis, we found that older people with pre-frailty/frailty are more likely to feel older than their chronological age only before adjusting for health-related variables. A few studies have reported that physical health is a strong predictor of subjective age [37, 38]. Reduced handgrip strength as a criterion of Fried frailty phenotype [3] was associated with older subjective age [30]. Previous studies have indicated that individuals with better self-rated health may feel younger [37, 39]. Self-rated health, while not a component of the Fried frailty phenotype, is also a marker of frailty [40]. Negative age stereotypes and social stigma may explain the effects of health on subjective age [9–11, 41]. Older adults are often considered worthless and incompetent [42]. Older adults with poor physical health (such as frailty populations) have more negative age stereotypes compared with healthy ones, which leads to relatively older subjective ages. Our study suggested that some health-related variables may mediate the predictive effect of frailty on subjective age, but the exact mechanism needs to be further explored.

From a clinical perspective, we need to pay attention to those older adults who feel older than their chronological age for their future frailty screening. It has been shown that subjective age is modifiable [24], and recent research suggests that standard cognitive and neuropsychological tests can influence older adults’ subjective age [43]. Intervention to modify subjective age may help prevent or delay frailty in later life. The relationship between subjective age and frailty needs to be investigated in further studies.

There are several limitations in this study. Although this study used a nationally representative sample, which is one of the strengths of our study, it included community-dwelling older adults only. Thus, results should be generalized with caution to other populations, such as nursing home residents and clinical samples. This study used a single question to measure subjective age. Some researchers have argued the concept of subjective age is multidimensional and cannot be measured by a single item [38]. However, there is also evidence that the single-item subjective age measurement is a robust method [44]. This study was a secondary data analysis and the covariates that could be adjusted for in the database were limited.

## Conclusions

To our best knowledge, this is the first study to investigate the bidirectional association between subjective age and frailty using a nationally representative longitudinal dataset, which has extended the current knowledge on subjective age and frailty. The present study found that older adults with higher subjective age were also more likely to be frailty. Future intervention programs to delay frailty progression should include strategies that may help older adults perceive a younger subjective age.

## Data Availability

The data sets analyzed in the current study are publicly available: NHATS (https://www.nhats.org/).

## Abbreviations

ADL: Activities of Daily Living
IADL: Instrumental Activities of Daily Living
NHATS: National Health and Aging Trends Study
U.S.: United States
IRB: Institutional Review Board
BMI: Body Mass Index
PHQ-2: Patient Health Questionnaire-2
GEEs: Generalized Estimating Equations
SD: Standard Deviation
ANOVAs: Analysis of Variance
OR: Odd Ratio
CI: Confidence Interval

## References

[1] Clegg A, Young J, Iliffe S, Rikkert MO, Rockwood K (2013). Frailty in elderly people. Lancet.

[2] Choi J, Ahn A, Kim S, Won CW (2015). Global prevalence of physical frailty by Fried’s criteria in community-dwelling elderly with national population-based surveys. J Am Med Dir Assoc.

[3] Fried LP, Tangen CM, Walston J, Newman AB, Hirsch C, Gottdiener J, Seeman T, Tracy R, Kop WJ, Burke G (2001). Frailty in older adults: evidence for a phenotype. J Gerontol A Biol Sci Med Sci.

[4] Morley JE, Vellas B, Van Kan GA, Anker SD, Bauer JM, Bernabei R, Cesari M, Chumlea W, Doehner W, Evans J (2013). Frailty consensus: a call to action. J Am Med Dir Assoc.

[5] Lang P-O, Michel J-P, Zekry D (2009). Frailty syndrome: a transitional state in a dynamic process. Gerontology.

[6] Marcucci M, Damanti S, Germini F, Apostolo J, Bobrowicz-Campos E, Gwyther H, Holland C, Kurpas D, Bujnowska-Fedak M, Szwamel K (2019). Interventions to prevent, delay or reverse frailty in older people: a journey towards clinical guidelines. BMC Med.

[7] Michel J-P, Cruz-Jentoft AJ, Cederholm T (2015). Frailty, exercise and nutrition. Clin Geriatr Med.

[8] Collard RM, Boter H, Schoevers RA, Oude Voshaar RC (2012). Prevalence of frailty in community-dwelling older persons: a systematic review. J Am Geriatr Soc.

[9] Palgi Y (2016). Subjective age and perceived distance-to-death moderate the association between posttraumatic stress symptoms and posttraumatic growth among older adults. Aging Ment Health.

[10] Hausknecht S, Low LF, O’loughlin K, McNab J, Clemson L. Older adults’ self-perceptions of aging and being older: A scoping review. The Gerontologist. 2020;60(7):e524–e534. 10.1093/geront/gnz153 31872232

[11] Weiss D, Lang FR (2012). “They” are old but “I” feel younger: Age-group dissociation as a self-protective strategy in old age. Psychol Aging.

[12] Kotter-Grühn D, Neupert SD, Stephan Y (2015). Feeling old today? Daily health, stressors, and affect explain day-to-day variability in subjective age. Psychol Health.

[13] Stephan Y, Sutin A R, Terracciano A. How old do you feel? The role of age discrimination and biological aging in subjective age. PloS one. 2015;10(3):e0119293. 10.1371/journal.pone.0119293 PMC4349738 25738579

[14] Choi NG, DiNitto DM (2014). Felt age and cognitive-affective depressive symptoms in late life. Aging Ment Health.

[15] Stephan Y, Sutin AR, Luchetti M, Terracciano A (2017). Feeling older and the development of cognitive impairment and dementia. J Gerontol B Psychol Sci Soc Sci.

[16] Stephan Y, Sutin AR, Terracciano A. “Feeling younger, walking faster”: subjective age and walking speed in older adults. Age (Dordr). 2015;37(5):86. 10.1007/s11357-015-9830-9 PMC5005834 26296609

[17] Stephan Y, Sutin AR, Terracciano A. Physical activity and subjective age across adulthood in four samples. Eur J Ageing. 2019;17:469–76. 10.1007/s10433-019-00537-7 PMC7752936 33381000

[18] Stephan Y, Caudroit J, Jaconelli A, Terracciano A (2014). Subjective age and cognitive functioning: a 10-year prospective study. Am J Geriatr Psychiatry.

[19] Stephan Y, Sutin AR, Terracciano A (2016). Feeling older and risk of hospitalization: evidence from three longitudinal cohorts. Health Psychol.

[20] Rippon I, Steptoe A (2015). Feeling old vs being old: associations between self-perceived age and mortality. JAMA Intern Med.

[21] Stephan Y, Sutin AR, Terracciano A (2018). Subjective Age and Mortality in Three Longitudinal Samples. Psychosom Med.

[22] Infurna FJ, Gerstorf D, Robertson S, Berg S, Zarit SH (2010). The nature and cross-domain correlates of subjective age in the oldest old: Evidence from the OCTO Study. Psychol Aging.

[23] Liang K (2014). The cross-domain correlates of subjective age in Chinese oldest-old. Aging Ment Health.

[24] Stephan Y, Chalabaev A, Kotter-Grühn D, Jaconelli A (2013). "Feeling younger, being stronger”: an experimental study of subjective age and physical functioning among older adults. J Gerontol B Psychol Sci Soc Sci.

[25] Shrira A, Bodner E, Palgi Y (2014). The interactive effect of subjective age and subjective distance-to-death on psychological distress of older adults. Aging Ment Health.

[26] Keyes CL, Westerhof GJ (2012). Chronological and subjective age differences in flourishing mental health and major depressive episode. Aging Ment Health.

[27] Spitzer N, Segel-Karpas D, Palgi Y. Close social relationships and loneliness: the role of subjective age. Int Psychogeriatr. 2019;1–5. 10.1017/S1041610219001790 31708003

[28] Stephan Y, Caudroit J, Chalabaev A (2011). Subjective health and memory self-efficacy as mediators in the relation between subjective age and life satisfaction among older adults. Aging Ment Health.

[29] Espinoza SE, Fried LP (2007). Risk factors for frailty in the older adult. Clin Geriatr.

[30] Hwang Y, Hong GS (2019). Predictors of subjective age in community-dwelling older adults in Korea. Geriatr Nurs.

[31] Bandeen-Roche K, Seplaki CL, Huang J, Buta B, Kalyani RR, Varadhan R, Xue Q-L, Walston JD, Kasper JD (2015). Frailty in older adults: a nationally representative profile in the United States. J Gerontol A.

[32] Rubin DC, Berntsen D (2006). People over forty feel 20 % younger than their age: Subjective age across the lifespan. Psychon Bull Rev.

[33] Zeger SL, Liang K-Y. Longitudinal data analysis for discrete and continuous outcomes. Biometrics. 1986;42(1):121–30. 3719049

[34] Hanley JA, Negassa A, Edwardes MDD, Forrester JE (2003). Statistical analysis of correlated data using generalized estimating equations: an orientation. Am J Epidemiol.

[35] Cui J, Qian G (2007). Selection of working correlation structure and best model in GEE analyses of longitudinal data. Commun Stat Simul Comput®.

[36] Buckinx F, Charles A, Rygaert X (2018). Own attitude toward aging among nursing home residents: results of the SENIOR cohort. Aging Clin Exp Res.

[37] Bergland A, Nicolaisen M, Thorsen K (2014). Predictors of subjective age in people aged 40–79 years: A five-year follow-up study. The impact of mastery, mental and physical health. Aging Ment Health.

[38] Kotter-Grühn D, Kornadt AE, Stephan Y (2016). Looking beyond chronological age: Current knowledge and future directions in the study of subjective age. Gerontology.

[39] Stephan Y, Demulier V, Terracciano A (2012). Personality, self-rated health, and subjective age in a life-span sample: the moderating role of chronological age. Psychol Aging.

[40] Gonzalez-Pichardo A, Navarrete-Reyes A, Adame-Encarnación H, Aguilar-Navarro S, García-Lara J, Amieva H, Avila-Funes J (2014). Association between self-reported health status and frailty in community-dwelling elderly. J Frailty Aging.

[41] Marquet M, Boutaayamou M, Schwartz C, Locquet M, Bruyere O, Croisier JL, Adam S (2018). Does negative information about aging influence older adults’ physical performance and subjective age?. Arch Gerontol Geriatr.

[42] Wurm S, Westerhof GJ (2015). Longitudinal research on subjective aging, health, and longevity: Current evidence and new directions for research. Annual Rev Gerontol Geriatr.

[43] Geraci L, De Forrest R, Hughes M, Saenz G, Tirso R (2018). The effect of cognitive testing and feedback on older adults’ subjective age. Neuropsychol Dev Cogn B Aging Neuropsychol Cogn.

[44] Westerhof GJ, Miche M, Brothers AF, Barrett AE, Diehl M, Montepare JM, Wahl H-W, Wurm S (2014). The influence of subjective aging on health and longevity: a meta-analysis of longitudinal data. Psychol Aging.

